# Supplementary material for: Getting More Out of Biomedical Documents with GATE's Full Lifecycle Open Source Text Analytics
Source: PLoS Comput Biol. 2013 Feb 7;9(2):e1002854. doi: 10.1371/journal.pcbi.1002854 (PMC3567135; doi:10.1371/journal.pcbi.1002854)
Supplement: Dataset S1 — GATE software. Dataset S1 bundled with this paper contains a distribution of GATE (or see http://gate.ac.uk/download/). (TGZ) [file pcbi.1002854.s001.tgz › plos-gate/doc/announce-7-0.html]

GATE Developer/Embedded Version 7.0 release (February 2012)


# GATE Developer/Embedded Version 7.0 release (February 2012)

GATE Developer/Embedded version 7.0 introduces a number of new features,
including:

- a rewritten plugin manager supporting the installation of new plugins from
  remote update sites directly from the GATE Developer GUI
- the first public release of the new JAPE Plus execution engine for JAPE
  grammars
- a new suite of tools for biomedical text processing
- improved support for right-to-left languages in the document editor

... and many more improvements and bug fixes. Full details can be found in the
changelog in the user guide.

The GATE Developer/Embedded package is available for download from
http://gate.ac.uk/download/.
